# The Vomiting of Pregnancy

**Published:** 1882-12

**Authors:** William B. Atkinson


THE VOMITING OF PREGNANCY. Read before the Philadelphia County Medical Society, September 13, 1882. BY WILLIAM B. ATKINSON, M.D.

Perhaps no condition of the pregnant woman is so full of annoyance, and at times so liable to lead to disastrous consequences, as the nausea which occurs particularly in the early months. Totally absent with some, with others it becomes a source of distress and danger, ceasing only with the expulsion of the fœtus or the death of the patient.

While the latter extreme is very rare, the constant spitting, nausea, or vomiting is frequently the accompaniment of a pregnancy, rendering the life of the woman a burden, and she only obtains a certain degree of comfort when, under the influence of anodynes, sleep secures for her a respite.

The limits of an introductory paper will not permit of any extended remarks beyond the strict line marked out by your business committee,—" The Prevention and Treatment of the Vomiting of Pregnancy."

Now, in order that we may be prepared to treat or prevent a disease, it is eminently necessary that we should understand its cause, its real nature. Undoubtedly this condition results from two causes. In the early stages, the nausea, etc., are due to sympathetic disturbance of the stomach. Later, we have added direct pressure upon and interference with the functions of that viscus.

Now, these causes act with greater or less power as they occur in a patient with an irritable stomach, one prone to be disturbed by the slightest irregularities in food or digestion, or in one who has an ostrich-like power which enables her to load the stomach and carry through to the bowels matters which can only be partially digested, and must eventually pass away as foreign bodies.

With the hope of making pregnancy so comfortable that the ordinary objection to this condition on the part of the woman may be greatly lessened, the entire prevention of nausea and vomiting has been proposed and sought for by many obstetricians.

We must not forget at this point that in earlier days, and even now to a limited extent, it was believed that a sick pregnancy was a healthy one, and many practitioners, when these symptoms were absent, endeavored to imitate them by the employment of ipecacuanha and similar drugs.

It is difficult to understand how to proceed to prevent a condition which has not presented itself, and which may never occur. For we find in practice that a large number of women never exhibitthe slightest nausea in any of their pregnancies and many others suffer so little that it is deemed of no moment.

We may, indeed, having other reasons to regard the woman as pregnant, advise her to avoid carefully all articles of food which are likely to give rise to irritable stomach, and also to observe care as to the regularity of her meals and habits. Beyond this we have no indications by which to be guided. The treatment of this trouble may then be considered under the heads of its relief when present, and efforts to prevent its return.

In the milder cases it is doubtful how much of the benefit is due to the remedy, and how much to the course of nature, in which the trouble would disappear spontaneously.

Many of the so-called cures which are so highly vaunted owe their supposed efficacy, in the successful cases, to the fact that little was needed to quiet an irritated stomach, irritated, perhaps, by carelessness or over-indulgence, which was intensified by the condition of the patient. It is only under this view that we can understand the wonderful effects of remedies as diverse as their names.

Therefore we may expect, and almost invariably obtain, relief in the great majority of cases from the use of sedatives and narcotics, as the bromides, chloral, morphia, carbonic acid water; from alkalies; from bitters, by their tonic action on the stomach, as gentian, calumba, quassia, nux vomica, etc.; from stimulants, as brandy, aromatic spirits of ammonia, champagne, the latter acting both by its stimulating quality and by the carbonic acid; from care in diet, carelessness in which frequently produces the earliest symptoms; from hygiene, change of locality, scenery, and occupation.

Those of us who attended the lectures of the late Prof. Charles D. Meigs can recall the convincing manner in which he showed us how to combat this trouble, by requiring the patient to take a cup of tea and a piece of toast while yet in bed, only rising sufficiently to rest upon the elbow while eating, and then to resume the recumbent position until the stomach had time to acquire tone to enable her to arise without the nausea.

It is from these trifling cases that we have constantly heralded the wonderful benefits to be obtained by the use of certain remedies, which soon lose their hold on the profession, and are only recalled as a curiosity in the literature of the medical art.

As such cases are of very frequent occurrence, and demand treatmant, we may mention those remedies which have proved most successful. Thus we have prussic acid in small doses; aconite of which the adminstration of a few drops of the tincture has been found of great benefit; the use of horseradish scraped fine, moistened with vinegar; arsenic, which is with many a favorite remedy; atropia or belladonna, combined with morphia; bismuth, the sub-nitrate, or, as preferred by some, the phosphate; calumba, highly extolled by Bartholow and others; carbolic acid, administered in drop doses; the oxalate of cerium, which at one time was regarded as a specific in these cases; chloral, which is usually prompt, and is best given in the form of an enema; chloroform and ether, either in small doses or by inhalation; the latter has proved useful when sprayed upon the spine; creasote, one of the earliest remedies; sulphate of copper, gr. iv. to water f℥j, five or six drops at a dose; hyoscyamia, which is claimed as effectual when all else has failed; iodine; ipecacuanha, which has recently been reported on by several observers, given in drop doses of the wine in a teaspoonful of water, repeated every hour; pepsin, lactopeptin, and their compounds; nux vomica, five to ten drops of the tincture, and recommended by Bartholow where the nausea is great, with little vomiting, in drop doses; the bromides of potassium and sodium, used by Busey in doses of thirty to sixty grains, dissolved in beef-tea, with the addition of brandy and laudanum, if required by the symptoms, and thrown into the rectum every four hours. Friedrich is tempted to regard the bromide of potassium as a specific in one to two grain doses daily.

Recently we have on most excellent authority, a preparation made of the inner coating of the gizzard of the chicken, dried and reduced to an impalpable powder. In our hands this has frequently acted with great promptness and efficiency. While we may readily, and generally do, succeed in obtaining positive relief for our patients by the employment of some one or more of the above remedies, yet we occasionally encounter a case which obstinately refuses to yield to any remedy by the mouth, everything being rejected almost as soon as it reaches the stomach.

In these instances the happiest results frequently follow medication by the rectum. Especially do we find this to obtain with chloral, which we have known to act speedily and pleasantly in a number of instances. Perhaps a better plan is the use of chloral, morphia, and belladonna or atropia, combined in a mucilage or in the form of a suppository. In several cases we have thus obtained a tolerance of food by the stomach, and thus we have relieved a threatened death by starvation.

In other cases we have obtained even better results by direct contact of these remedies with the os, and even within the cervix of the uterus.

In one case where, owing to the animal instincts of the husband, the nausea and vomiting were constantly reproduced, we each time succeeded in speedily checking it by passing a suppository of morphia, belladonna, and hyoscyamus up to the os, and keeping it closely applied.

Tanner employs suppositories made of belladona, gr. iij ; hyoscyamus, gr. x ; and iodide of lead, gr. viiss.

Injections, both rectal and vaginal, are advocated by many, and Dr. Greene has succeeded with warm olive oil, after failing with warm water. Cold to the epigastrium, small pieces of ce swallowed or passed into the rectum or vagina, have proved serviceable.

As might be expected electricity has its advocates, and Gaillard Thomas esteems it higher than any other remedy. A broad, flat electrode, made by stitching a flat sponge to sheet rubber, he fixes by means of adhesive plaster on the epigastrium, and a similar one under the spine, the patient lying on her back. A gentle current is passed, and continued for ten or even twenty-four hours.

Da Venezia, in a similar case, after all else had failed, used a faradic current of moderate strength, one rheophore being applied to the side of the neck along the vagus, the other to the epigastrium. The patient was relieved at once, and after the fourth application was cured.

J. Marion Sims employs caustic to the os, even when the parts appear perfectly healthy. He claims the best results from this method.

It would appear that this treatment is that which in obstinate cases promises the best results. Especially is it indicated when an examination reveals erosion or other disease of the os uteri. Originally, the sole dependence was upon upon nitrate of silver, but equally good results are obtained by the glycerole of iodine.

Tannin, carbolic acid, in short a great variety of remedies capable of relieving engorgement, erosion, or whatever evil condition may be present, have been found to act promptly in relieving the nausea, etc.

From the remarks of Dr. Sims and from the results in general practice it would appear as though all that is required is a means of producing an impression of a positive nature at the real seat of the affection, the os uteri. Hence we find severe cases are frequently at once terminated by ordinary anodyne or astringent injections, applied so as surely to produce their effect upon the os uteri.

While there is no doubt as to the safety of these applications properly employed, it is much to be questioned whether the same may be said of the plan proposed and carried out by some practitioners abroad,—that is, the dilatation of the os with the finger. No doubt such a method would at once relieve the nausea, but at the same time it might be anticipated as extremely likely to result in an abortion. For this reason I would hesitate as to its employment until every other remedy had failed, and the question had arisen whether we should not sacrifice the child to save the mother.

I earnestly believe that the question of premature delivery will rarely, if ever, occur when the treatment which I have so roughly sketched has been properly employed.

In this connection we may allude to the cases, though rare, where the husband has been the victim of nausea, while the pregnant wife was enjoying her usual health. Here we certainly cannot regard it as the result of sympathy between the womb and the stomach.

Before closing the subject of treatment I may mention that Pinard obtained immediate relief in several obstinate cases by the employment of inhalations of oxygen. I am not aware that it has been tried in this country.

A valuable aid, by allowing complete rest for the stomach, is the employment of rectal alimentation. Dr. H. F. Campbell has employed this plan in a number of instances, and with most gratifying results. Twice each day he injects very slowly and gently about eight ounces of beef-tea or some similar nutritious fluid. The advantages are complete rest for the stomach while nutrition is readily maintained. In the intervals between the injections a full goblet of water not quite cold was twice given, so as to supply the requisite amount of fluid.

In conclusion, permit me to sum up what I think is the duty of the practitioner in these cases:

The most complete rest of body and mind.

The avoidance of all forms of diet save those of easy digestion and assimilation.

The relief of the early symptoms by some one of the articles mentioned under the head of medication. Unless prompt relief is obtained, the use of chloral, morphia, belladonna, or hyoscyamus, or their combination, by the rectum or by the vagina. In the latter case it is important that we should first carefully cleanse away the discharge usually found clinging to the os, and maintain them there by the usual methods.

This failing, apply to the os and cervix, if need be, the glycerole of iodine or the nitrate of silver, and follow this by an application of the anodynes, as before.

If the vomiting is now great, abandon the stomach as a depot for food, and employ rectal alimentation solely. In each injection we may include with the nutrient chloral to aid in procuring complete rest.

To relieve the intense thirst which is generally present, we may allow the patient to swallow at intervals small lumps of ice, or to drink iced carbonic acid water, which is now so readily obtained from the siphon. Of course, just sufficient of this should be taken to relieve the throat at the momeut.

I do not consider the dire alternative of induced abortion, as such a procedure rarely becomes necessary, and should only be employed after the most careful deliberation, and after a council of physicians have decided it to be imperative.—*Philadelphia Medical Times.*

---

SEX AND RACE.—In most European nations the proportions of male births to female is as 105 to 100. Among the Jews, however, the proportion rises to 110 or 120 to 100. This is thought to be due to the fact that marriage is earlier among the Jews.—*Medical Record.*

